# Report of the Committee of the House of Commons on the Petition Respecting the Fever Institution

**Published:** 1804-08-01

**Authors:** 


### Report of the Committee of the House of Commons *on the* Petition *respecting the* Fever Institution.

THE Committee, to whom the Petition of the several persons whose names are subscribed thereto, being Members of the Society for bettering the Condition of the Poor, was referred; and who were empowered to report the matter of the said petition as it should appear to them, with their observations thereupon, to the House; have, pursuant to the order of the House, examined the matter to them referred; and have agreed to the following Report.

Sir Walter Farquhar, M. D. having been examined, informed your Committee, that to his knowledge and experience rience the infectious fever was very prevalent in London, particularly among the poorer parts in confined situations; that, from information, which he repeatedly had, and on which he could rely, he had reason to believe that more than three thousand persons have annually died of it in and about London. That there could be no doubt that a house for receiving patients in the fever, would prevent the spreading the infection; particularly, if the houses from which the patients are removed, were properly cleaned and white-washed, and still more so if the bed cloaths, and also the patient's cloaths were burnt immediately. That he had known the most fatal consequences follow from the neglect of this precaution, even in the instance of a single article coming from an infected person. That he did not conceive that an establishment, on a sufficient scale to answer those beneficial purposes, could be formed in the metropolis without public aid, and had no scruple to say that he believed it impossible. That the infection is likely to be generally communicated, by the necessary intercourse subsisting between servants and other members of large Families, with persons in the lower classes of life, among whom the infectious fever more commonly prevails. That he has known repeated instances of its being so communicated to persons in the higher classes of life, particularly among children, and has found it necessary to lay it down as a rule in families where he is consulted, that the nurses should never have any communication with friends in their own houses, or with others they may casually meet. That there is good reason to hope, that by the judicious, diligent, and persevering efforts of such an institution as has been alluded to, that the infectious fever may be very considerably diminished, and even in the end almost entirely extirpated from the metropolis. And that the extinction of the infectious fever in the metropolis, would very greatly tend to prevent its taking place in those parts of the country which have a communication with it, more especially as the fever generally remains lurking in the constitution for some time before it prevents the patient from travelling or following his usual occupations. That it is of great importance in the cure of these fevers, to attend to them in an early stage; and general attention cannot be given to patients so infected in the wide extent of the metropolis, except by the establishment of an institution upon a considerable scale, and very carefully directed, so as to be capable of receiving all the infected persons who may apply. That the poor when in their their own houses, labouring under the infectious fever, must always be attended by their friends with hazard; and though some peculiar constitutions may escape, in general the attendance must be highly dangerous.

Maxwell Garthshore, M. D. having been examined, also informed your Committee, that an institution to prevent the spreading of contagious malignant fevers, has been established within these three years in Gray's Inn Lane Road, which, although upon a very contracted scale, previous to February last received 325 fever patients, of whom 289 have been dismissed cured, and the houses of many of them, and others, purified, white-washed, and cleansed; 29 died, and seven then remained in the house. That he does not think the present establishment sufficiently extensive, to remedy the evil of the infectious fever in the metropolis. That a public institution would afford means both of preventing and curing the infectious fever, which cannot be practised in the houses of the poorer classes of society; but that he is of opinion that a public institution, of sufficient extent to remedy the evil in the metropolis, cannot be established by private subscription unassisted by public aid. That it is of great importance that the infectious fever should be attended to in its early stages; and that he believes that not one case was ever taken into this institution in an early stage, which was not soon recovered, it being a disorder the prevention and cure of which are perfectly well understood. That the infectious fever has prevailed for a long course of years in some of the crouded parts of the metropolis, in a more or less degree, according to seasons and circumstances.

Thomas Bernard, Esq. having been examined, likewise informed your Committee, that the London Fever Hospital in Gray's Inn Lane was established in May 1801, and a House of Recovery opened in Gray's Inn Lane in February 1802. That great exertions have been made in every possible way to obtain subscriptions, and yet the present funds are not equal to the maintaining that small house on the present scale. That the whole sum which has been raised by donations and subscriptions, has amounted to £.4378, the annual expences of the house are between £.600 and £.700, and that the house is not adequate to one-tenth part of the patients who want relief, as it contains only sixteen beds. Finding that this establishment will not be adequate to the purpose, the society have begun raising a fund of £.3000, and £.1600 has already been subscribed, upon condition that parliament would grant such a sum, as, together with those subscriptions, would prove effectual to the desired object.

Upon the whole, your Committee find that the infectious fever has been long prevalent among the poor of London, and that above 3000 persons have, on an average, annually died of it within the bills of mortality.

This evil appears to your Committee to have continued for want of some regular system being adopted to check its progress, and to purify the houses and other buildings in which it has prevailed. In May 1801, an Institution for the Cure and Prevention of Infectious Fevers in the Metropolis was established by the Society for bettering the Condition of the Poor; and in February 1802, a London House of Recovery was opened, in which, although it contains no more than sixteen beds, upwards of 320 fever patients have already been cured.

This establishment has been hitherto supported by the contributions and subscriptions of individuals, and nearly the whole of £.4000 so collected has been expended upon it. The extent of the institution appears to your Committee insufficient for the attainment of its object; and although great exertions have been used, it has not been found practicable to raise, by private subscriptions, funds large enough to increase or support it, more especially as it is essentially necessary to destroy the cloaths of the patients, and purify the houses in which they have been attacked by the complaint. In the expectation that, towards an object of so much national importance, aid might be afforded by the legislature, a sum of £.1600 has been subscribed by several individuals, upon the condition of such assistance being first received. The Institution cannot, therefore, have the benefit of this bounty, without it is also supported by Parliament; and, upon the whole, it appears to your Committee, that the object of the petition is of the highest importance, and that the proposed Institution would be productive of great public advantage.

